# Epoxy vs. Calcium Silicate-Based Root Canal Sealers for Different Clinical Scenarios: A Narrative Review

**DOI:** 10.3390/dj12040085

**Published:** 2024-03-25

**Authors:** Hadas Azizi, Avi Hadad, Dan Henry Levy, Joe Ben Itzhak, Hyeon-Cheol Kim, Michael Solomonov

**Affiliations:** 1 Department of Endodontics, Israel Defense Forces (IDF) Medical Corps, Tel Hashomer, Ramat-Gan, Israel; 2 “Bina” Program, Faculty of Dental Medicine, Hebrew University of Jerusalem, Jerusalem, Israel; 3 Department of Conservative Dentistry, School of Dentistry, Dental Research Institute, Pusan National University, Yangsan 46241, Republic of Korea; golddent@pusan.ac.kr

**Keywords:** clinical situation, epoxy resin-based sealers, calcium silicate-based sealers, root canal obturation

## Abstract

This study aimed to review the considerations for choosing a suitable sealer according to various endodontic scenarios. An electronic search of PubMed, Scopus, and the Web of Science was undertaken for the keywords of ‘sealer choosing’, ‘appropriate sealer’, ‘suitable sealer’, ‘sealer for clinical scenario’, and ‘sealer for clinical situations’. However, the literature review revealed a lack of studies with practical clinical recommendations regarding the choice of appropriate endodontic root canal sealers for particular clinical situations of root canal treatment. Therefore, a narrative review was undertaken under the basis of the characteristics of an epoxy resin-based sealer (ERS) versus a calcium silicate-based sealer (CSS). Based on the evidence found through the review, the choice of an appropriate sealer in a variety of clinical scenarios was proposed. An ERS is recommended for one-visit non-vital cases, teeth with periodontal involvement, cracked teeth, and internal root resorption without root perforation. A CSS is recommended for vital or non-vital cases in multiple visits, teeth with internal root resorption with perforation or internal approach for external cervical resorption, teeth with open apices, and teeth with iatrogenic aberrations.

## 1. Introduction

Root canal obturation is a one of the critical determinants of the success of endodontic treatment [1]. To achieve the goal of canal obturation, root canal sealers should be used along with a gutta-percha (GP) for a fluid-tight or hermetic seal throughout the canal, including the apical foramen and canal irregularities [2].

As an ideal sealer, it should have fine particles to mix well with liquid, not shrink upon setting, not discolor the tooth structure, be bacteriostatic, must not encourage the growth of bacteria, be insoluble in tissue fluids, and mut be tissue tolerant. Moreover, it should provide adhesion to the canal wall when set, form a hermetic seal, and be radiopaque [3].

Numerous types of endodontic sealers are available at present. Presently, no sealer satisfies all the criteria perfectly. Nevertheless, AH Plus (Dentsply DeTrey, Konstanz, Germany) is an epoxy resin-based root canal sealer (ERS) considered the gold standard for its physicochemical properties [4]. On the other hand, calcium silicate-based root canal sealers (CSSs) have become popular in the endodontic practice over the last decade [5,6,7].

The present article reviews the considerations for choosing suitable root canal sealers according to different clinical situations.

### 1.1. Literature Search and Scope of the Review

An electronic search of PubMed, Scopus, and the Web of Science was undertaken with the keywords including ’sealer choosing’, ‘appropriate sealer’, ‘suitable sealer’, ‘sealer for clinical scenario’, and ‘sealer for clinical situations’. However, the literature review revealed a lack of studies with practical guidance and recommendations regarding the choice of optimal endodontic root canal sealers for particular clinical endodontic situations. Therefore, a narrative review of ERS versus CSS characteristics and the proposed sealer in varied clinical scenarios was undertaken.

### 1.2. Epoxy Resin-Based Sealers (ERSs)

ERSs, one of the well-investigated types of sealers, are characterized by high toxicity immediately after mixing [8,9]. They present antibacterial activity during setting; as they are set, they are inherent visible [10,11]. They also have a high bond strength, both to the dentine and the GP [12,13,14], low solubility [15,16,17], and dimensional stability with relatively low shrinkage (0.2%) [18]. The smear layer reduces the ERS’s bond strength to dentinal walls [19,20].

According to the above-mentioned properties, these sealers have preferred properties in thin (0.05 mm) and thick (0.3 mm) films [21]. Therefore, it can be interpreted that they use various cold and warm techniques. The warm technique can be associated with a steep learning curve and requires suitable equipment [22]. Moreover, preheating of the epoxy resin-based sealer up to 120 °C induces a delayed and prolonged secretion of pro-inflammatory cytokines TNF-α and IL-6 [23]. Stainless-steel spreaders and pluggers used inappropriately during these compaction techniques can result in vertical root fractures [24].

To date, ERSs have performed well clinically and in laboratory tests over the decades [25]; thus, they are considered predictable sealers with favorable results.

### 1.3. Calcium Silicate-Based Sealers (CSSs)

CSSs have recently gained popularity and have been incorporated into the practitioner’s armamentarium [6,7]. CSSs demonstrate good biocompatibility and an ability to penetrate dentinal tubules from good flowability [6,7,26,27,28]. They release calcium hydroxide during the setting reaction [29], which exhibits an antimicrobial effect [10,30,31] that also contributes to the sealer’s biocompatible and bioactive (mineralization) nature [32,33].

The ISO 6876 specification recommends that the dimensional change should not exceed 1.0% shrinkage or 0.1% expansion [34]. Due to their calcium hydroxide releases, CSSs have a more consistent magnitude of dimensional change across different conditions, reaching up to 20% [4,32,35,36]. CSSs are known to be non-dissolvable in a solvent. Therefore, they should be mechanically removed when endodontic retreatment is required [37]. Retreatment may be feasible if a poor obturation technique is performed or other circumstances occur [38].

A CSSs’ expansion can be speculated as a factor that enhances the quality of the obturation. However, at the same time, it can be considered a predisposing factor to crack formation, especially in non-round root canals. Furthermore, calcium hydroxide release can result in a constantly high pH that can damage dentin properties [39]; after 2 to 3 months, the dentin’s strength may be reduced [40].

CSSs have a hydrophilic nature. Moisture in dentinal tubules catalyzes the setting reaction of CSSs [26,41]; however, according to the manufacturer, the use of paper points for canal drying is recommended. The dentinal tubules content may be different depending on the pulp statuses (vital, non-vital, or retreatment cases). The patient’s age [42], as well as pathological or iatrogenic factors over the individual’s lifespan, such as carious lesions or deep restorations [43], attrition or abrasion [44], occlusal trauma [45], periodontal disease and treatment [46], and orthodontic treatment [47], can alter the dentin’s water content, increase collagen modifications, and elevate reactionary dentin deposition.

According to the above-mentioned properties, these sealers have preferred properties in a thick film. Therefore, this provides the single-cone technique’s most accessible and quickest obturation [48] in round canals and the lateral passive technique in oval canals.

The microhardness values of CSSs are reduced in an acidic environment and thus have more porous and less crystalline microstructures. Therefore, it is questionable whether these sealers are suitable in inflamed areas with a low pH value [49,50,51]. Other drawbacks of CSSs are that they might cause tooth discoloration [52,53], especially due to contamination with blood [54]; GP–sealer interfaces are more frequent in CSSs [55]. Moreover, they remain moderately cytotoxic over six weeks [41]. 

## 2. Clinical Situations for Different Preferable Root Canal Sealers

ERSs and CSSs have both demonstrated clinical success in clinical studies and are deemed acceptable sealers [5,56,57]. The unique properties of each sealer, identified through basic research, facilitate a clearer understanding of their effectiveness across various clinical scenarios.

### 2.1. Single vs. Multiple Appointments in Non-Vital Cases

Single-visit root canal treatment has become a common practice and offers some advantages [58,59]. However, understanding the biological aspects of performing a single-visit endodontic treatment in non-vital cases is essential. A CSS is susceptible to low pH, which can interfere with its setting and mechanical properties. Thus, the inflammatory condition in non-vital teeth may not be appropriate for its use. In these cases, ERSs can be used. Treating non-vital cases over multiple visits, using calcium hydroxide as an intra-canal medicament, offers antibacterial effects. This facilitates the dissolution of lipopolysaccharides and raises the pH level. This is an optional scenario without known limitations for using either ERS or CSS sealers. However, the removal of calcium hydroxide may be accomplished to varying degrees [60,61]. Hence, CSSs have a greater adaptation to the canal wall [62,63], probably due to interactions between the calcium hydroxide produced during the sealer’s setting to the calcium hydroxide’s remnants on dentinal walls.

### 2.2. Cracked Tooth

**Figure 1:**
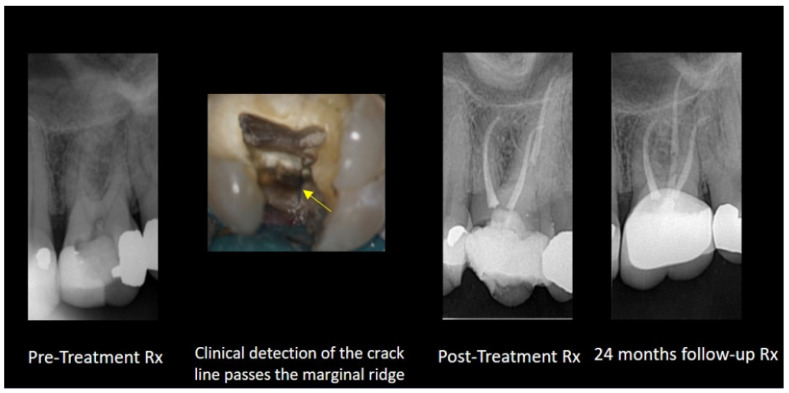
Maxillary first molar with a crack (yellow arrow demonstrates the crack line as the cavity is accessed). The tooth is obturated with the single-cone technique with GP and ERS. X-ray images of pre- and post-treatments and from a two-year follow up.

The pulpal and periapical status of a cracked tooth depend on the extent of the crack and its symptoms’ duration. When the crack extends into or is close to the pulp, ingress bacteria and their by-products can cause irreversible pulpitis, which can progress to pulp necrosis and subsequently apical periodontitis [64]. It can be speculated that when endodontic treatment is required in this specific clinical scenario, minimally invasive instrumentation should be preferred to minimize the taper and canal enlargement, as well as the absence of the use of a spreader or plugger with pressure. The benefits of the single-cone technique with ERSs is optional. The potential expansion of CSSs might be less appropriate in these cases. Figure 1 presents a case of a cracked tooth that is obturated with an ERS.

### 2.3. Root Canal Treatment for Teeth in Close Proximity to Anatomic Structures

A difference in the distance of the apices to the inferior alveolar nerve, mental foramen, and maxillary sinus exists [65,66,67,68]. The relationship between anatomic structures and teeth apices can serve as another factor that can influence the clinicians’ preference for one sealer over the other, since sealer extrusions were observed in more than 47% of cases treated with a calcium silicate-based sealer [5]. In cases presenting a close proximity to the anatomic structure with or without apical resorption, the use of ERSs should be preferred.

### 2.4. Resorptions

**Figure 2:**
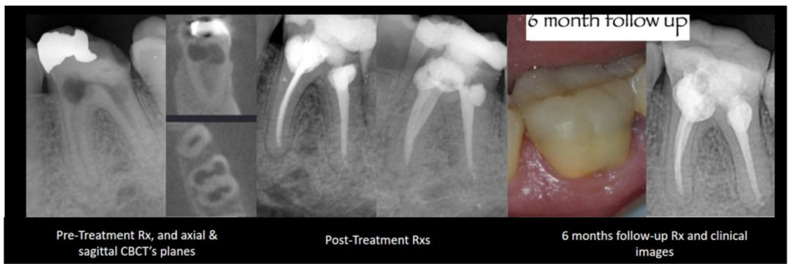
Mandibular first molar with two internal root resorption lesions without perforation that was obturated with GP and ERS. X-ray images of pre- and post-treatments and from a 6-month follow up.

**Internal root resorption without perforation:** Frequently, the dentin walls adjacent to an internal resorptive lesion, advanced yet without perforation, are thin; the root canal’s round-to-oval widening or ballooning-out appearance can also be observed [69]. Hence, a CSS may not be the most suitable option in such instances, given its expansion, the impact of calcium hydroxide on dentin’s mechanical characteristics, and the stress distribution resulting from its thickness and stiffness. Figure 2 presents a case of internal root resorption without perforation that was obturated with an ERS.

**Figure 3:**
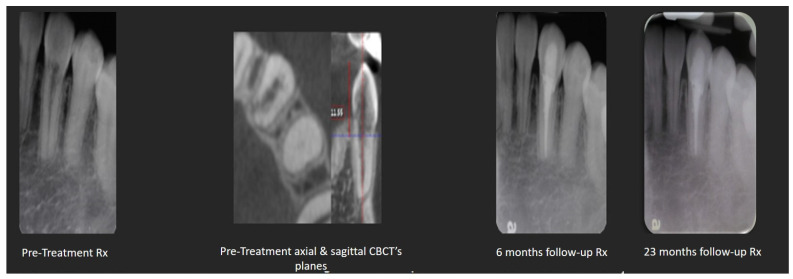
Mandibular canine with external cervical resorption (with a narrow subcrestal entry point) obturated with GP and TSS. X-ray images of pre- and post-treatments and from a 6- and 23-month follow-up sessions.

**Figure 4:**
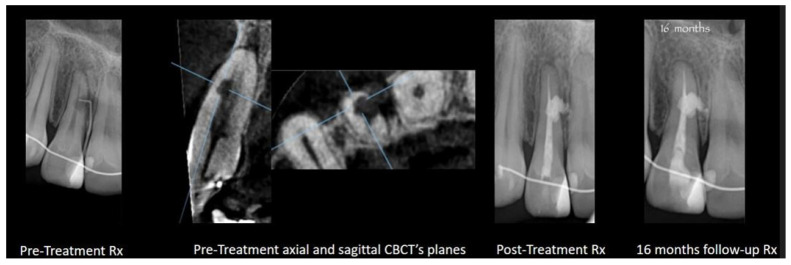
Maxillary lateral incisor with external cervical resorption (with a wide subcrestal entry point) obturated with GP and TSS. X-ray images of pre- and post-treatments and from a 16-month follow up.

**Internal root resorption with perforation and internal approach for external cervical resorption:** Treatment for advanced internal root resorption involving perforation and an internal approach for external cervical resorption can involve the use of a CSS. Its suitability for these scenarios is attributed to its biocompatibility, flowability, and the released calcium hydroxide. Biocompatibility is crucial, especially when the material extends to periradicular tissues, as anticipated in these cases. The release of calcium hydroxide contributes to a prolonged increase in pH, interfering with the activity of osteoclasts in the resorptive tissue. Additionally, the flowability ensures that the sealer adapts to resorptive dentinal defects. Figure 3 and Figure 4 present cases of external cervical resorption with subcrestal entry points that are obturated using a CSS.

### 2.5. Non-Divergent Open Apex

Classic apexification, a one-visit mineral trioxide aggregate (MTA) plug, and revascularization are the treatment options for pulpless teeth with an open apex [70]. These treatment options require special equipment, such as a dental operating microscope and MTA carrier, as well as a high level of operation endodontic skills. Yet, general practitioners worldwide seek to provide quality treatment in these cases. Thus, a new simplified approach based on CSSs was developed [71]. Compared to traditional sealers, the significant advantage of a CSS is its biocompatibility, which can play an important role in open-apex cases.

**Figure 5:**
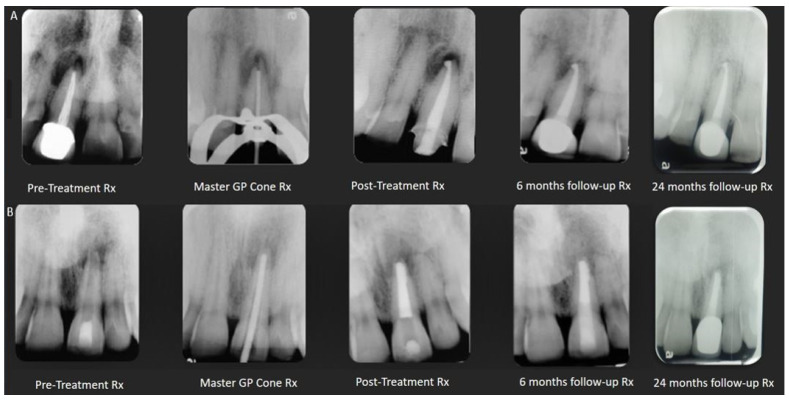
(**A**,**B**): Maxillary anterior teeth with non-divergent open apices that were obturated with GP and TSS. X-ray images of pre-treatment, GP master placement, post-treatment, and from 6-month and two-year follow-up sessions [71].

The method: after proper chemo-mechanical preparation, the canal is dried, and a GP master cone is selected. The operator must opt for the largest cone or customized cone that stops 1 mm short of the working length (if a stop is not achieved, another treatment option needs to be selected). The cone is coated with a CSS and inserted into the canal, 1 mm short of the working length. Accessory GP cones, coated with the CSS, can be added passively without a spreader. Heat is then applied with a warm plugger to cut the excess GP, and the GP is compacted lightly with a plugger. Figure 5A,B present cases that are obturated in this manner.

### 2.6. Iatrogenic Aberrations

**Figure 6:**
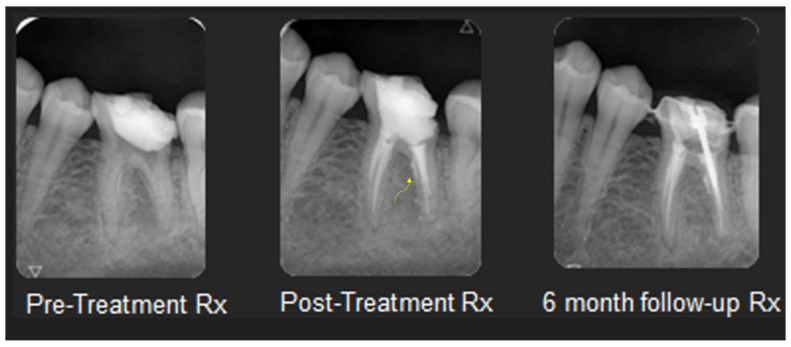
Mandibular first molar with a strip perforation that is obturated with GP and TSS. The yellow arrow indicates the sealers’ flow through the perforation on the post-treatment X-ray. X-ray images of pre- and post-treatments and from a 6-month follow up.

**Strip perforation:** The complete penetration of a root canal wall due to excessive lateral tooth structure removal during canal preparation is defined as a strip perforation [72]. It usually occurs in curved roots or roots with surface invaginations. Treatment options for this scenario can include a two-step technique involving endodontics and surgery [73] and MTA as an artificial barrier [74]. In addition, using an internal [75] or modified matrix [76] might be necessary to reconstruct the root’s outer shape and facilitate the adaptation of MTA. Special equipment, such as a dental operating microscope and MTA carrier, might be required. The operator should be skilled in this practice as well. Obturation with a CSS can provide a simple way to handle this condition. Its flowability enhances performing an ordinary root canal obturation; so, in that manner, the sealer occupies the strip perforation site. Over-extruded material probably has no negative influence due to its biocompatibility [5,77]. Furthermore, follow-up sessions demonstrate that the extruded material in the peri-radicular tissue can be dissolved or washed out [5]. Figure 6 presents a case with a strip perforation that is obturated with a CSS.

**Inaccessible iatrogenic root canal perforation:** Perforations beyond the root canal curve might not be advisable with a dental operating microscope; hence, many of the ways to handle perforations are irrelevant in these cases. As for strip-perforation cases, a CSS can provide a simple way to handle this condition.

## 3. Endo-Perio Lesion

Belli et al. [78] reported that stresses at the apical end of the root increase with increases in lesion dimensions. Considering that MTA-based sealers or an MTA plug can create more stress when there is periodontic involvement or a true combined lesion, a CSS should be used with caution when there is a primary endodontic disease with periodontal involvement due to the sealer’s stress distribution. Thus, in these cases, ERSs should be used.

### Ergonomic Considerations

Preserving maximum tooth structure in order to optimize the biomechanical behavior of endodontically treated teeth [79] leads to the abandonment of the traditional endodontic cavity and the performance of conservative, constructed, truss and ninja endodontic cavities [80,81].

The normal average mouth-opening results in healthy children and adolescents (7 to 19 years old) are 35–38 mm [82], 45 mm in 19-to-70-year-old patients, and 38 mm in patients over 70 years old [83]. The tendency to use minimally invasive endodontics combined with limited mouth opening can present difficulties during the endodontic procedure. Thus, it can negatively affect dental ergonomics aspects and endodontic treatment quality, especially in the molar region.

**Figure 7:**
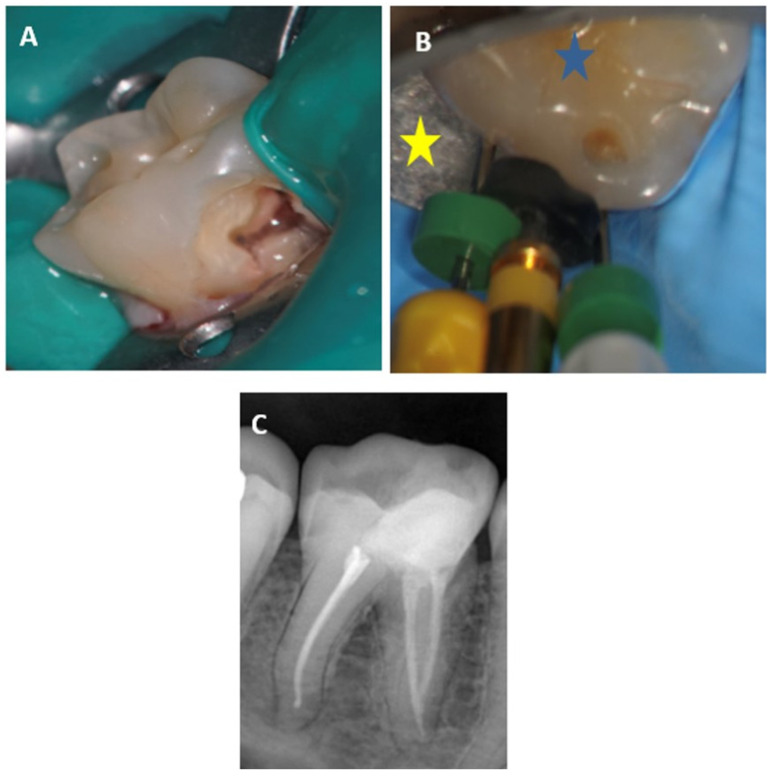
Mandibular molar with a buccal access cavity: (**A**)—the access cavity after the pulp was exposed; (**B**)—the files are inserted into the canal *(blue star*—the occlusal plane of the tooth; *yellow star*—the buccal aspect of the dental clamp); (**C**)—final X-ray image of the root canal, after obturations with GP and TSS.

Nickel–titanium-controlled memory wire instruments that allow pre-bending [81] and the use of the single-cone technique with a CSS can be used to address these ergonomic factors. Figure 7 presents a case with a buccal access cavity, due to limited mouth opening, which is obturated with a CSS.

## 4. Discussion

Endodontics is far from being a simplistic practice. Once the diagnostic phase is completed, the practitioner must carefully select the suitable treatment option after considering the patient’s self-perception and health. Endodontic equipment and materials are extensive and the practitioner should consider various terms and conditions and adjust the selected materials according to each clinical case.

As there is no single ideal sealer available, new products with various technology have been developed. For example, the idea of using macromolecules with antibacterial features has evolved; the addition of nanoparticle macromolecules to sealers to gain antibiofilm properties has also been considered [84,85]. Innovative ideas are not permanent since the intended results have not been achieved. Although there is no ideal root canal sealer, the epoxy resin-based root canal sealer is still considered the gold standard. On the other hand, CSSs have gained popularity in endodontic practice over the last decade. The advantages and drawbacks of each sealer may dictate their applications. Practitioners should be skilled in estimating the clinical characteristics of each case so they can choose one sealer over the other depending on the case.

However, no original studies suitable for this purpose have been carried out. Thus, the present review is appropriate to attempt to interpret different endodontic scenarios for practical clinical suggestions regarding those sealers within the limitations of the existing studies: an ERS is recommended for one-visit non-vital cases, teeth with periodontal involvement, cracked teeth, and internal root resorption without root perforation. A CSS is recommended for vital or non-vital cases over multiple visits, teeth with internal root resorption with perforation or internal approach for external cervical resorption, teeth with open apices, and teeth with iatrogenic aberrations. Moreover, the clinician should also take into account the existence of various CSS materials with diverse chemistries, including different percentages of calcium silicate, which can potentially impact their clinical performance [86,87].

Although a narrative review is not a systematic review and it is subjective, there are no formal rules for selecting studies or standard statistical methods for combining studies. Therefore, readers need to remember that authorial bias may or may not be present when reading and evaluating a narrative review.

In light of the contemporary advancement of endodontic sealers, future research endeavors should aim to provide more comprehensive insights into the optimal sealer selection for specific diagnostic scenarios, including conducting a comprehensive analysis of the genes regulated by each root canal sealer [88].

The potential benefits of conducting prospective randomized clinical trials cannot be overstated, as they offer a robust foundation for evidence-based decision making in sealer choices. These trials could reveal the long-term outcomes and clinical efficacy of different sealers in various clinical contexts, helping practitioners make informed choices based on the unique situation of each patient.

Further studies, such as a prospective randomized clinical trial, if possible, should be carried out to investigate the sealer suitable for each diagnosis. Meanwhile, the clinician’s awareness is essential.
